# Electroencephalography-Based Depression Detection Using Multiple Machine Learning Techniques

**DOI:** 10.3390/diagnostics13101779

**Published:** 2023-05-17

**Authors:** Amel Ksibi, Mohammed Zakariah, Leila Jamel Menzli, Oumaima Saidani, Latifah Almuqren, Rosy Awny Mohamed Hanafieh

**Affiliations:** 1 Department of Information Systems, College of Computer and Information Sciences, Princess Nourah bint Abdulrahman University, Riyadh 11671, Saudi Arabia; 2 Department of Computer Science, College of Computer and Information Sciences, Riyadh 11442, Saudi Arabia; 3 Department of Computer Science, College of Computing in Al-Qunfudah, Umm Al-Qura University, Makkah 24382, Saudi Arabia

**Keywords:** major depressive disorder (MDD), electroencephalogram (EEG), convolutional neural network, feature extraction, deep learning, depressive disorder

## Abstract

The growth of biomedical engineering has made depression diagnosis via electroencephalography (EEG) a trendy issue. The two significant challenges to this application are EEG signals’ complexity and non-stationarity. Additionally, the effects caused by individual variances may hamper the generalization of detection systems. Given the association between EEG signals and particular demographics, such as gender and age, and the influences of these demographic characteristics on the incidence of depression, it would be preferable to include demographic factors during EEG modeling and depression detection. The main objective of this work is to develop an algorithm that can recognize depression patterns by studying EEG data. Following a multiband analysis of such signals, machine learning and deep learning techniques were used to detect depression patients automatically. EEG signal data are collected from the multi-modal open dataset MODMA and employed in studying mental diseases. The EEG dataset contains information from a traditional 128-electrode elastic cap and a cutting-edge wearable 3-electrode EEG collector for widespread applications. In this project, resting EEG readings of 128 channels are considered. According to CNN, training with 25 epoch iterations had a 97% accuracy rate. The patient’s status has to be divided into two basic categories: major depressive disorder (MDD) and healthy control. Additional MDD include the following six classes: obsessive-compulsive disorders, addiction disorders, conditions brought on by trauma and stress, mood disorders, schizophrenia, and the anxiety disorders discussed in this paper are a few examples of mental illnesses. According to the study, a natural combination of EEG signals and demographic data is promising for the diagnosis of depression.

## 1. Introduction

A frequent mood illness called depression can result in a constant feeling of melancholy, a loss of interest, and memory and attention problems. Cognitive impairment and long-lasting, profound affective depression are common in depressed patients. In addition, paranoia and illusions may occur in certain people in severe instances [1]. As a result, it is crucial to diagnose depression when it is still treatable and can even save a patient’s life [1,2]. The mechanisms behind protracted unpleasant moods and depression are currently the subject of intense research into the human brain.

A scale-based interview conducted by a psychiatrist is the most common technique for diagnosing depression. EEG coherence is a strong indicator of integrated neuronal correlation when analyzing the linearly dependent relationships between the bandwidths of EEG signals collected from brain areas or working electrodes [3]. This measure generates a symmetrical two-dimensional matrix. The presence of high coherence between two EEG signals indicates coherent neuronal oscillations, inferring interconnection between neural populations. Low convergence, on the other hand, demonstrates independent activity. EEG coherence has proven to be a highly effective method for analyzing the brain activity of individuals with depression [3], Alzheimer’s disease (AD) [4] and Parkinson’s disease [5] using EEG coherence. The outcomes of the existing approaches for diagnosing depression, however, depend on the psychiatrist’s expertise and involve a lot of work.

Furthermore, because of the stigma associated with the condition and its nature, depressed people are less inclined to seek care. As a result, many individuals with depression receive incorrect diagnoses and inadequate care, delaying their recovery. Therefore, a growing area of research is discovering practical and reliable ways to identify depression. With the latest innovations in sensor and mobile technologies, analyzing physiological data for diagnosing mental diseases opens up a brand-new opportunity for a precise and objective tool for anxiety identification. Along with much other clinical information, the EEG exhibits deep personal human cognitive function [6,7]. The EEG recorded the monotonous, spontaneous electrical impulses of cells in the brain on the scalp. Since the discovery of the monkey brain and the first recording of the human EEG signal, scientists have investigated the association between brain function and mental disorders utilizing EEG data [8].

**Figure 1:**
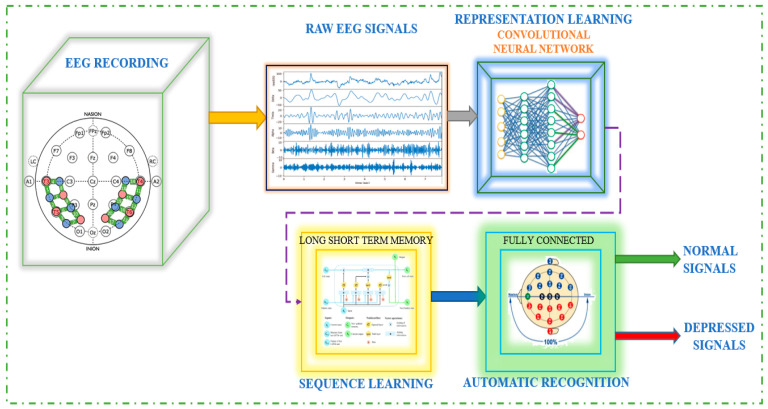
An Illustration of the EEG-Based Automatic Depression Detection System.

The EEG-based depression detection system is depicted in Figure 1, where raw EEG signals processed via CNN follow EEG recording. The signal is then sent to LSTM, also known as sequence learning, and finally to automated recognition.

The current widely used method for examining functional brain interconnection employs network analysis assessment to transform a functional brain matrix into a gradient. After accomplishing a categorization of the structural features of the graph, the clustering coefficient and attribute path length, two index values that interpret a chart and correlate to the two significant aspects of functional brain entity, such as functional splitting and connectivity [9], are utilized to distinguish between people suffering from neurological abnormalities and normal individuals. These two indices can also capture the network’s significant attributes accurately. Random network topology and small-world network architecture distribution have been demonstrated repeatedly in Alzheimer’s disease, schizophrenia, and depression. By demonstrating that AD is characterized by a loss of small-world network properties, [10] showed that the characteristic path length in AD patients was substantially longer than that in healthy controls. In schizophrenia, [11] discovered that the small-world network structure was randomized. According to one EEG study, MDD adjusts the topological organization of the cognitive system implicated in sleep, as demonstrated by the deficit of additional features [12].

Furthermore, [10] found that the brain networks of depressed patients during sleep are more similar to the random section of the reconfiguring spectrum. In contrast, the visual cortex networks of healthy controls are comparable to the scale’s ordered amount. Although abnormalities in brain function connections have been discovered in MDD, the brain functional network model of chronic depression is still unidentified. As a result, we used graph theory to examine the operational brain network of individuals afflicted with clinical depression.

DNNs have lately seen tremendous popularity in vector image, video, and text-based recognition tasks [13,14]. CNNs are used in the best current architecture processing techniques for image and video data because of their advantages in handling 2D input data [15]. CNN performs well in biological picture categorization, and the features it learns are frequently superior to hand-crafted features. According to research by [16], an ensemble system of custom-made and known features can improve CNN’s performance when categorizing biological images. Studies have employed various techniques to translate EEG signals into visual representations for neural signal categorization. One, in particular, combines 1D CNNs and stacked autoencoders with the short-term Fourier transformation approach to categorize EEG motor imagery signals [17].

EEG studies could be utilized to effectively comprehend the mechanisms underlying brain function, human cognitive abilities, the symptoms of frontal cortex ailments, and the field of the Brain–Computer Interface (BCI), which has notably gained a great deal of attention [18]. When equated with computed tomography (CT) and magnetic resonance imaging, EEG has a higher temporal resolution, lower costs of maintenance, and a more functional operation mode. As a possible consequence, EEG was recommended as a non-invasive procedure to research cognitive behavior [19] and other illness depression, such as chronic fatigue [20], seizure disorders, and insomnia [21], as an objective physiological tool for data consolidation. EEG has also been used to diagnose mental illnesses like depression, psychosis, and anxiety [22,23]. Additionally, depression always comes with irregular brain activity and blatant emotional swings because it is a mental condition with clinical signs like significant depression and slow thinking. EEG can therefore identify these strange events to monitor brain activity. The frequency of the EEG signal can be divided into several wave bands based on their frequency ranges. These include delta (0.5–4 Hertz), theta (4–8 Hertz), alpha (8–13 Hertz), beta (13–30 Hertz), and gamma (30–50 Hertz or higher). Delta waves are typically associated with deep sleep or unconsciousness; they are prevalent in stages 3 and 4 of sleep and are the predominant rhythm in newborns up to one year of age. Theta waves are observed to be more prominent during internal focus, meditation, prayer, and spiritual awareness. These waves are known to represent a transitional state between wakefulness and sleep and are associated with the subconscious mind. While it is considered atypical for theta waves to be present in fully awake adults, their presence is a natural occurrence in children up to 13 years of age. The alpha wave peak occurs at 10 Hz, and a healthy production of alpha waves is known to promote mental flexibility, coordination, and a sense of relaxation. When in this state, individuals can work efficiently to complete tasks at hand. Dominance of alpha waves is generally associated with feelings of calmness and comfort. Alpha waves have been linked to the connection between awareness and the subconscious mind. Typically, calm adults exhibit this primary rhythm, which is present for most of their lives. After the age of thirteen, it tends to dominate resting EEG traces. The beta wave is a normal rhythm commonly observed in individuals who are alert or anxious, as well as those with their eyes open. This state is associated with analytical problem-solving, decision-making, and processing information about the surrounding environment. It is the most common brain state during waking hours when an individual is actively listening and thinking. Gamma waves, which have the highest frequency, are associated with cognitive processing, attention, and perception [24].

**Figure 2:**
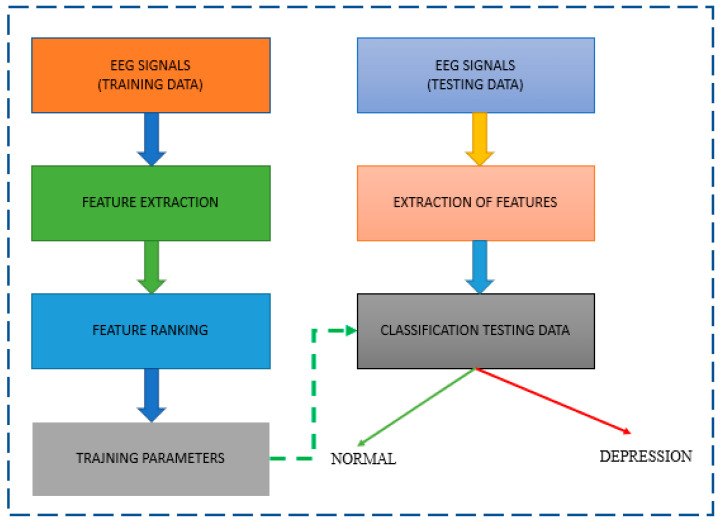
Block diagram of a system for EEG-based diagnosis of depression.

A typical approach for classifying normal and depressive EEG signals is shown in Figure 2. EEG studies of depression often use the properties of EEG signals to analyze the data. The local feature extraction module, also known as the signal processing module, comes before the LSTM and the classification module, which all proceed to the CNN. The successful development of a multifaceted, three-electrode EEG monitoring system by Intelligent Solutions Lab [25] is part of the current ability to contribute to this field. It was used to build a database of depressed patients and healthy controls.

MODMA, a multi-modal open dataset for analyzing mental illnesses, collects EEG signal data. The EEG dataset contains data from an advanced wearable 3-electrode EEG collector for widespread applications and a standard 128-electrode elastic cap. Three locations are used to store EEG data. As a result, the research has concentrated on analyzing a pervasive EEG-based depression detection system using cutting-edge data processing methods and machine learning.

The auditory tones used as the external stimuli. These tones were presented through headphones to both the healthy control group and the group of patients diagnosed with major depressive disorder. To ensure that the experiment was conducted in a consistent manner, the auditory stimuli were presented in a passive listening paradigm. During this paradigm, the participants were instructed to listen attentively to the sounds presented without any active response or task. This approach allowed us to isolate the brain responses to the auditory stimuli without any confounding factors that could arise from a specific task or cognitive demand. The auditory tones used in our study had a duration of 100 ms, which is a standard duration used in ERP studies. The inter-stimulus interval between the tones was 1000 ms. This interval was used to ensure that the auditory stimuli were presented in a controlled and consistent manner, allowing us to measure the brain’s response to each tone in isolation. The use of a consistent and controlled presentation of stimuli is critical in ERP studies, as it allows for the reliable measurement of the brain’s response to external stimuli.

For the ERP analysis, we selected several local peaks including N1, P2, N2, and P3, as these components have been previously shown to be sensitive to depression-related alterations in brain function. The amplitude and latency measures of these peaks were used to compare the differences between the two groups. N1, P2, N2, and P3 are common event-related potential (ERP) components that are often used in neurophysiological research. N1 (Negative 1) is a negative deflection that occurs approximately 80–150 ms after the onset of a stimulus. It is thought to reflect early sensory processing and attention allocation to the stimulus. P2 (Positive 2) is a positive deflection that occurs approximately 150–250 ms after stimulus onset. It is thought to reflect higher-order cognitive processing and attention, such as identifying and categorizing the stimulus. N2 (Negative 2) is a negative deflection that occurs approximately 200–300 ms after stimulus onset. It is thought to reflect cognitive processes related to stimulus evaluation, including working memory, attention, and decision-making. P3 (Positive 3), also known as the P300 or the “oddball” response, is a positive deflection that typically occurs 300–500 ms after stimulus onset. It is thought to reflect cognitive processes related to stimulus evaluation, including memory updating and response preparation. Both the mean and maximum amplitudes of specific time windows for each local peak, as well as the latency of each peak from stimulus onset, were determined to identify differences in brain function between healthy controls and patients with major depressive disorder. The following EEG signals in the resting state with 128 channels are taken into consideration in this project to support this research:

- Event-related potentials in response to external stimulation were recorded over 128 channels; 24 patients had major depressive disorder, while 29 persons in the healthy control group did not.
- In resting-state 128-channel recordings, 24 persons with major depressive disorder and 29 without the condition were found.
- Twenty-nine healthy control subjects and 28 people with major depressive disorder were found in 3-channel resting-state recordings, as detailed in the section below.

The purpose of the work is to use EEG data analysis to create an algorithm that can automatically identify depression tendencies. The complexity and non-stationarity of EEG signals, as well as the unique variations that may have an impact on the generalizability of detection systems, are discussed in the study. The suggested methodology integrates demographic information such as age and gender with EEG signals to increase the precision of depression diagnosis. The study used machine learning and deep learning methods to automatically identify people with depression. The usage of wearable EEG technology and open datasets in practical applications is also covered in the article. The study’s ultimate objective is to aid in the early and accurate identification of depression, which might improve patient outcomes.

Section 2 is devoted to discussing associated works in the organization of this review. Section 3 discusses data collection, while Section 4 discusses methodology. Section 5 contains the provided results. Section 6 includes a discussion. The conclusion is addressed in Section 7 at the end.

## 2. Literature Review

This section reviews the studies that have looked at EEG signals and deep learning techniques for diagnosing and predicting depression patients. Ref. [7] reviewed research that used EEG data to identify the two types of depression, bipolar disorder (BD) and MDD, using neural network and deep learning techniques. It searched among papers published over the previous ten years using a variety of search engines and a mix of different keywords, then took some valuable information from those. The fact that this review classified exploited datasets, techniques for analyzing or extracting features, and algorithms in the publications was one of its strong qualities [11,12]. It also creates many tables to exhibit the extracted data and allow comparisons between them in different ways. Only about five articles, as indicated, especially for MMD diagnosis, were considered an apparent fault in this research because it needed to employ a significant number of publications to review. Additionally, the journals must explain their general concept and method more. The review by [26] focused on studies using deep learning techniques to investigate mental diseases, including depression. The four primary areas of this study were the detection of mental illness using clinical data, genetic data in disease diagnosis, analysis of various datasets, and social media data to estimate the risk of mental illness. Only three studies that dealt with depression diagnosis or prediction employed the electroencephalogram dataset type out of the selected papers that were published up through April 2021. The examined datasets were wholly represented in this study. An in-depth discussion was also given about the opportunities and difficulties that could result from using each dataset. However, because the review was comprehensive and focused on a wide range of mental illnesses, several studies on deep learning for depression diagnosis and prediction using EEG data were briefly discussed. CNNs have recently been used to investigate the possibility of EEG encoding and decoding. Ref. [11] proposed a parallel linear CNN to capture dynamic and static energy identifiers. In [10], CNN was utilized for features extracted from epileptic intraoperative EEG signals. In [27], EEG signals were transformed into multi-spectral images and decrypted using recurrent CNN. Ref. [10] used a 13-layer CNN model to detect depression. In light of the entitlements of CNN, this work describes 1D CNN to retrieve spatiotemporal representations of EEG signals. Convolutional neural networks (CNNs) have recently been recognized as an essential and reliable deep learning methodology. Recently, the method has expanded its employment in biomedical signal and image processing problems due to its notable success in computer vision [28]. Researchers have also concentrated on creating a CNN-based computer-aided diagnosis system for the medical industry. Ref. [29] developed a CNN model for extracting EEG data and characterizing the signal as predicted, preictal, or convulsion, with an overall accuracy of 89.8%. Ref. [30] provided a 1D CNN to structure standard and pathological EEG data instantly and discovered a 21.10% classification error. Refs. [10,31] recently developed CNN models with eleven and thirteen layers to recognize depressed patients using EEG signals. Ref. [32] created a one-dimensional CNN-based model with 91.33% accuracy to detect cardiac arrhythmia from long-term ECG signal segments. In a comparison study of Alzheimer’s disease diagnoses performed via three distinct NN models, the FFNN, the block-based neural network, and CNN, CNN was the best classifier [33].

Ref. [34] proposed using a kernel eigen-filter-bank typical spatial pattern to extract characteristics from the EEG of twelve patients suffering from severe depression and twelve normal individuals. The study used the leave-one-subject-out cross-validation assessment method to achieve an SVM classifier recognition rate of 80%. Ref. [35] estimated the power spectral density in multiple bandwidths (theta, beta, and alpha) as well as the entire band of the EEG signal to categorize forty depressive patients and forty healthy subjects.

A review of studies [4] examined how EEG signals and various classifiers could be used to monitor issues like emotion identification and identify neurological diseases like depression. Only four publications were from an earlier period, and most of the papers were published between 1999 and 2020, using various sources, including journals, books, conferences, and theses. Only about ten articles related to the diagnosis of depression were considered. This provided a comprehensive comparative assessment of the techniques and data used in publications separated into separate regions, such as artifact removal, types of extracted features, dimensionality reduction, feature selection, and clustering algorithm algorithms [36]. Based on their method concerning the collection gathered, numerous adopted datasets were summed up and are presented as general and local recognition categorizations. Additionally, this included details on functional neuroimaging methods. However, because it covered so many fields of research on mental health issues, the method needed to treat each one more thoroughly. Table 1 shows a list of past paper references with methodology used and results.

According to the literature evaluation, the present research gap is that just a few studies have used EEG data and deep learning algorithms to diagnose and predict depression. While some studies have used EEG signals to diagnose or predict depression, the majority of studies have focused on other mental health issues. Furthermore, few studies have extensively discussed the difficulties associated with using EEG data and deep learning techniques for depression diagnosis and prediction. More research into the most appropriate methods for feature extraction from EEG signals is also needed to improve the accuracy of depression diagnosis and prediction. Finally, further study is needed to examine the efficacy of various deep learning approaches for diagnosing and predicting depression using EEG data. The suggested technique overcomes constraints in using EEG data to diagnose depression. To capture the complexity and non-stationarity of EEG data, multiband analysis is performed. To account for individual differences, demographic parameters such as gender and age are incorporated in the modeling. Machine learning and deep learning approaches are utilized to improve accuracy and efficiency in automated depression diagnosis. The approach employs a vast and diversified dataset (MODMA) spanning a variety of mental diseases, as well as traditional and wearable EEG collectors, which improves the system’s generalizability and resilience.

## 3. Dataset

The multi-modal open dataset MODMA, which is used for the investigation of mental disorders, is where EEG signal data are obtained. Data from both a conventional 128-electrode elastic cap and a cutting-edge wearable 3-electrode EEG collector for widespread applications are included in the EEG dataset.

A 128-electrode elastic cap is a common EEG recording equipment item used in research and clinical settings. It consists of a cap that is fitted over the participant’s scalp, with 128 electrodes placed at specific locations according to the International 10-10 system. These electrodes detect electrical signals generated by the brain and transmit them to an amplifier, which amplifies the signals and converts them into digital data for further analysis. A wearable 3-electrode EEG collector is a newer type of EEG recording equipment that is designed for widespread applications. It typically consists of a small device that is worn on the forehead or behind the ear, with three electrodes that are placed in contact with the skin. These electrodes detect electrical signals generated by the brain and transmit them to a wireless receiver or a smartphone app, which records and analyzes the signal. It is noted that the eyes were closed during EEG recording to reduce any potential visual artifacts caused by eye movement. Lighting levels were also kept constant to minimize the effect of visual stimuli on brain activity. Moreover, additional methods for artifact correction, such as time-domain signal filtering or spatial filtering techniques, were commonly used to further improve the quality of EEG signals.

Table 2 summarizes the characteristics of three different experiments that were conducted on participants with major depressive disorder and healthy controls. For EEG, there are three datastores.

1. When External Stimulation is Used:
  - Age range: 16 to 52 years; 128-channel event-related potential recordings; 24 major depressive disorder participants; and 29 healthy control subjects.
  - Contains demographic information and psychiatric evaluations.
2. Under Rest:
  - Age range: 16 to 52 years; 128-channel recordings of participants in their resting states; 24 major depressive disorder patients and 29 healthy controls; demographic information; and psychological evaluations.
3. Three channels when at rest:
  - Age range: 16 to 56 years; 3-channel resting-state recordings; 26 major depressive disorder participants; and 29 healthy control subjects.
  - Contains demographic information and psychiatric evaluation.

This project considers 128-channel resting state EEG signal recording data. The inclusion criteria for participants in the MODMA dataset includes individuals between the ages of 18 and 55 years old with normal or corrected-to-normal vision and a primary or higher education level. For participants diagnosed with major depressive disorder (MDD), the diagnostic criteria of Mini-International Neuropsychiatric Interview (MINI) must be met, and the Patient Health Questionnaire-9item (PHQ-9) score must be greater than or equal to 5. MDD patients must not have received any psychotropic drug treatment in the last two weeks. Control participants should not have a personal or family history of mental disorders. All participants must provide written informed consent. The inclusion criteria ensure that the study sample is representative of the population of interest and that the study results are generalizable to the intended population.

The exclusion criteria for participants in the MODMA dataset include individuals with mental disorders or brain organ damage, serious physical illness, or severe suicidal tendencies for MDD patients. Participants with a personal or family history of mental disorders are excluded from the control group. In addition, participants who have abused or been dependent on alcohol or psychotropic drugs in the past year, women who are pregnant or lactating, or taking birth control pills are excluded from the study. These exclusion criteria ensure that participants are healthy and have not been exposed to any substances that could affect their brain function. The criteria also help to minimize any potential confounding factors that could influence the results and increase the internal validity of the study.

The Analysis of Variance (ANOVA), a statistical analysis, was carried out to compare the mean age of two groups, and the outcome indicated that there was no significant distinction between the mean age of the two groups. The results of the ANOVA revealed that there was no significant difference in the mean age between the depression group and healthy control group. The *p*-value was greater than 0.05, indicating that any differences in EEG signals between the two groups were unlikely to be solely caused by the difference in age between them. Therefore, it can be inferred that the lack of age difference between the two groups suggests that the differences in EEG signals were more likely due to the presence of depression in the depression group rather than an age difference between the two groups.

### 3.1. Data Visualization

**Figure 3:**
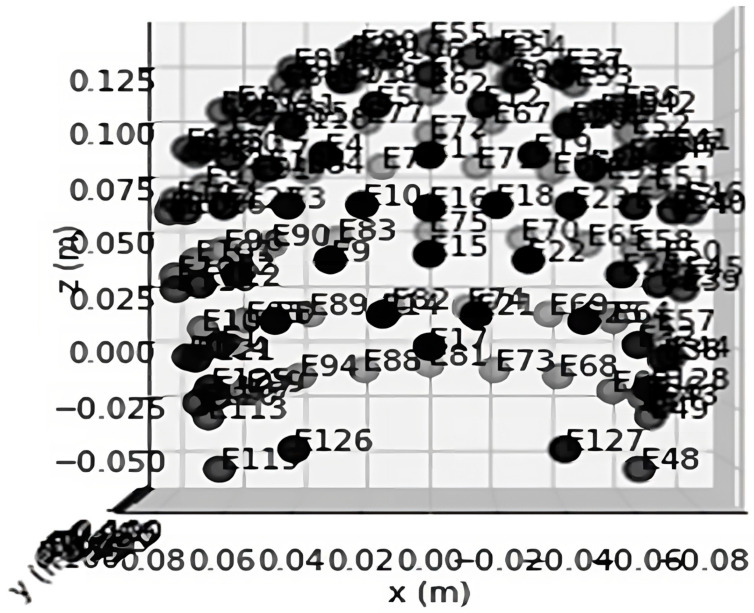
Architecture of 128-Channel Headset Data Visualization.

**Figure 4:**
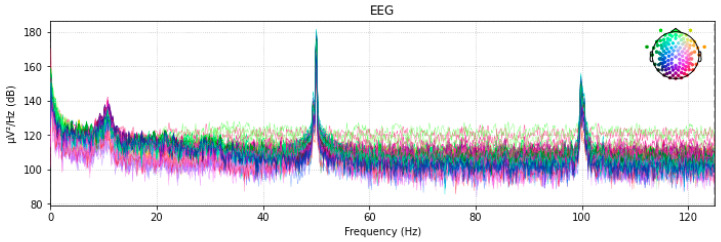
A 128-Channel Power Spectral Distribution.

Typically, an EEG machine has a number of electrodes. The electrodes are positioned on the patient’s scalp, and after extracting voltage, they transform it into signal data. For instance, if there are n electrodes, each electrode will produce a time series of voltage values. Different parts of the brain have different voltage levels. The architecture of a typical 128-channel headset is shown in Figure 3:

Figure 4 displays the 128-channel voltage for the resting state power spectral distribution at a sampling frequency of 250.

**Figure 5:**
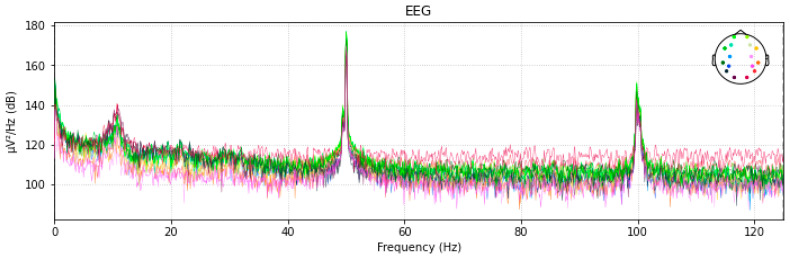
A 16-Channel Power Spectral Distribution.

Frequency information for 128 channels of EEG signals is shown in the figure below. Figure 5 displays the power spectral distribution for 16 channels.

AC is responsible for the increase in power at frequency = 50,120. These spikes are viewed as signal noise that will be eliminated in the next part.

### 3.2. Pre-Processing

#### 3.2.1. Artifact Correction and Re-Referencing

Artifact correction and re-referencing are important steps in EEG data preprocessing to improve the quality of EEG signals and remove unwanted artifacts. In the context of this study, artifact correction was likely performed to remove any electrical noise or artifacts caused by muscle movement or eye blinks.

One common method for artifact correction is Independent Component Analysis (ICA), which separates EEG signals into independent components that correspond to different sources in the brain or outside the brain, such as muscle activity or eye movement. By identifying these components, the artifacts can be isolated and removed from the EEG signals.

Re-referencing is a process of changing the reference electrode to improve the signal-to-noise ratio and enhance the detectability of EEG signals. In the context of this study, the EEG signals were likely referenced to a common reference electrode or a reference-free method was applied. This is done to eliminate or minimize the impact of spatially distributed electrical activity that is unrelated to the underlying brain activity of interest.

#### 3.2.2. Noise Removal

**Figure 6:**
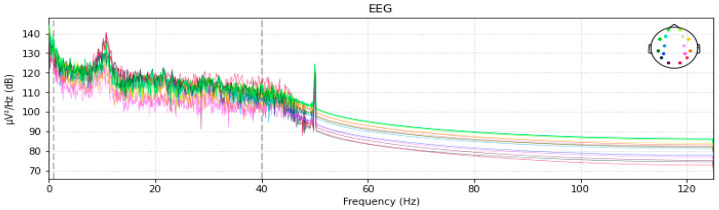
Noise Removal through Band-pass Filter.

The power spectral distribution spikes are eliminated using a bandpass filter with a filter size of 50 Hz. Since we cannot process signals directly for feature extraction, the function built additionally turns the EEG signal into a NumPy array in addition to eliminating these spikes. After using the band pass filter, the smoothed power spectral is shown in Figure 6.

#### 3.2.3. Feature Engineering

After the noise is removed from the EEG signals, they are transformed to a NumPy array, then feature engineering is used to extract valuable features from the data that will be used to distinguish between the EEG power spectrum of a healthy person and that of a mentally ill person. Following are two different kinds of features:

1: Linear features;

2: Nonlinear features.

Following are the linear features which are given as: power at alpha, power at beta, power at delta, power at theta, mean amplitude, median amplitude, maximum amplitude, minimum amplitude. The explicit EEG features used in the analysis are linear and nonlinear features. The linear features include power at different frequencies such as alpha, beta, delta, and theta. The amplitude of power signals including mean, median, maximum, and minimum are also used as linear features. The nonlinear features used in the analysis are spectral entropy and singular-value deposition entropy. The Pandas data frame contains all the extracted features, including linear and nonlinear features.

Power can be seen at various frequencies, including alpha, beta, delta, and theta. Amplitudes of power signals include mean, median, maximum, and minimum. The Pandas data frame contains the features that were extracted. The linear features are shown in Table 3.

#### 3.2.4. Non-Linear Features

The supplied EEG signal datastore is used to extract two nonlinear features: spectral entropy and singular-value deposition entropy. These two characteristics demonstrate how much valuable information is lost from the signal. The retrieved nonlinear features are shown in Table 4.

#### 3.2.5. Feature Allocation and Visualization

All of the features in this step are collected into a single data frame and saved in a CSV file that will be used in the following section. Table 5 in the citation below displays the Pandas data frame, which stands for the feature datastore.

#### 3.2.6. Visualization of Linear Features

**Figure 7:**
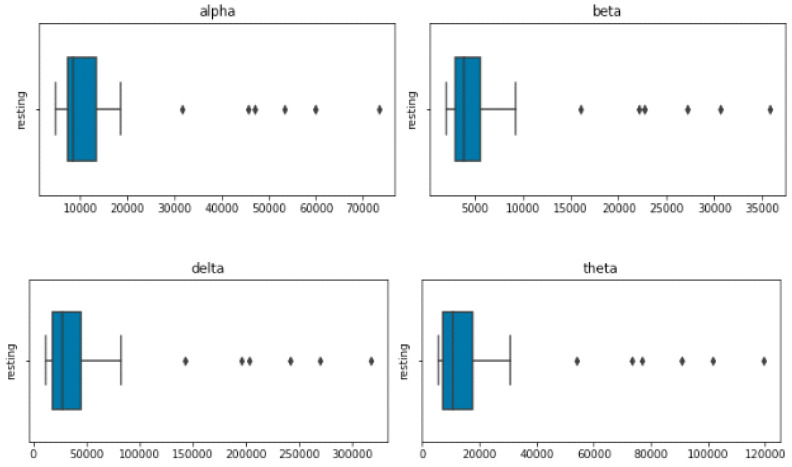
Visualization of Linear Features.

The terms “transformation” and “function” both refer to something that takes in a number and produces a number, such as f(x) = 2xf(x) = 2xf, where x is the input number and x is the output number. However, despite the fact that we frequently visualize functions using graphs, the phrase “transformation” is frequently used to imply that you should instead see a thing moving, stretching, squishing, etc. Consequently, the translation of the function f(x) = 2xf(x) = 2xf, left parenthesis, x, and right parenthesis, equals 2, x, gives us the multiplication-by-two video above. The number line’s point one is moved to where two begins, two to where four begins, etc. Figure 7’s Visualization of Linear Features section displays four EEG features—delta, theta, alpha, and beta—that were retrieved.

#### 3.2.7. Visualization of Power Spectral Features

**Figure 8:**
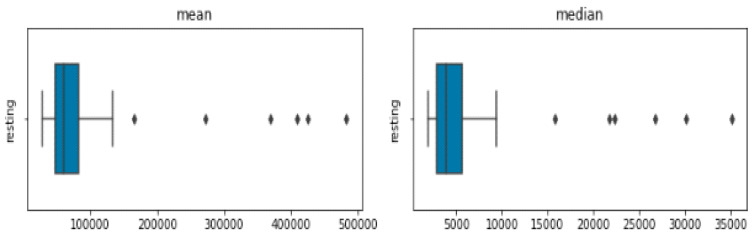

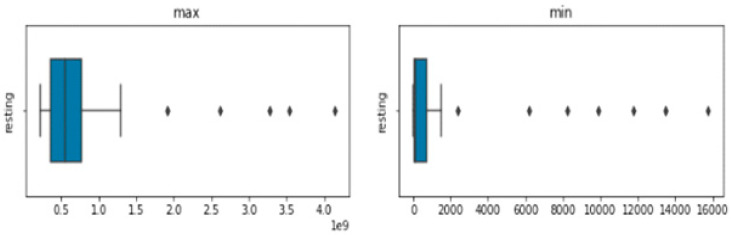
Power Spectrum Visualization.

The frequency and power characteristics of a signal are extracted using the block called spectral features. Unwanted frequencies can also be filtered out using low-pass and high-pass filters. Figure 8 shows the visualization of four power spectral features, including the minimum, maximum, median, and mean.

## 4. Methodology

### 4.1. Model Development

At first, characteristics were extracted from EEG data from several patients while they were at rest. Along with the patient’s condition and demographic information, these data are merged. The patient’s current state of health will serve as the response, and the retrieved attributes will be employed as a predictor. Major depressive disorder and healthy control are the two basic categories into which the patient’s condition can be divided. Additional MDD subtypes include the following six:

- Obsessive-compulsive disorders;
- Addictive disorder;
- Trauma and stress-related disorder;
- Mood disorder;
- Schizophrenia;
- Anxiety disorder.

All the demographic data kept in the Pandas data frame are displayed in Table 6 below.

The features are integrated with this data frame, as shown in Table 7, to generate the training datastore.

### 4.2. Data Preprocessing and Pre-Operation

The columns for age, gender, IQ, and serial number are removed from this data frame as well as any null entries. The target columns and predictor columns for classification are selected after the dataset has been analyzed. The remaining columns of the feature are set as predictor columns, with the MDD column set as the target column. Data are subjected to cross-validation using 10-fold validation.

#### 4.2.1. Label Datastore

The label datastore is displayed below in Table 8.

#### 4.2.2. Feature Datastore

Table 9 displays the feature datastore.

#### 4.2.3. Classification Model

For the classification of each MDD based on the features that were taken from the EEG data, three models were created.

- XGBOOST;
- Random Forest;
- 1D CNN model.

### 4.3. XGBoost

A distributed, scalable gradient-boosted Decision Tree (GBDT) machine learning framework is called Extreme Gradient Boosting (XGBoost). Parallel tree boosting is a feature of the best ML library for regression, classification, and ranking problems. Table 10 below lists the XGBoost parameters. According to the findings, feature optimization combined with the XGBoost algorithm improves classification accuracy. A number of features are extracted from the EEG brain signals in this work, and the set of features is then optimized utilizing the correlation matrix, information gain computation, and recursive feature removal approach.

**Figure 9:**
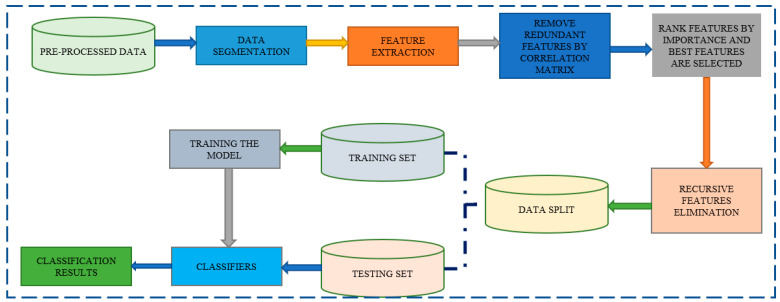
Overview of XGBoost Algorithm.

Figure 9 depicts the overall operation of the XGBoost method, which preprocesses the data before segmenting it, extracting features, and creating a correlation matrix. The data splitter separates it into training sets and testing sets once it has received the data. The classification outcome is presented by the XGBoost classifier last.

- Features of XGBoost
  - Parallelization: The model is trained over several CPU cores.
  - Regularization: XGBoost offers a range of regularization penalties in order to prevent overfitting. Penalty regularizations result in successful training, which enables accurate generalization of the model.
  - Non-linearity: XGBoost can recognize and learn from non-linear data patterns.
  - Cross-validation: Pre-installed and readily available.
  - Scalability: Thanks to distributed servers and clusters like Hadoop and Spark, XGBoost can handle large amounts of data.

### 4.4. Random Forest Model

The Random Forest method’s ensemble of Decision Trees is constructed from a data sample selected from a training set and a replacement sample known as the bootstrap sample. The RF model’s parameters are shown in Table 11.

#### Model Parameters

**Figure 10:**
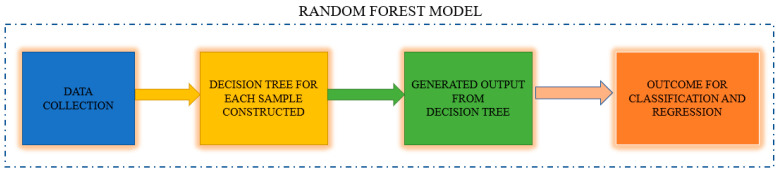
Random Forest Model.

The steps of the Random Forest algorithm are as follows and as shown in Figure 10:

- Step 1: The Random Forest technique uses n randomly chosen records from a data collection of k records.
- Step 2: A distinct Decision Tree is constructed for each sample.
- Step 3: Each Decision Tree will generate an output.
- Step 4: The final outcome for classification and regression is assessed using a majority vote or an average.

### 4.5. 1D CNN Model

The creation of a neural network is a very iterative process that calls for adjusting a number of hyperparameters to maximize the output. Additionally, trying out other architectures is part of it. Here, we will begin by constructing a sequential CNN. It will include our classification layer, two convolution layers, one dropout layer, one max pooling layer, one flatten layer, and one dense connected layer. Table 12 lists the parameters for the 1D CNN model, whereas Table 13 lists the hyperparameters.

- **Model Architecture**

**Table 12:** Parameters for 1D CNN Model.

Layer	Properties	
Input layer-Conv 1D	Input shape = (11,144)	Output shape = (1)
Conv 1D	Kernal size = 11	Activation = relu layer
Drop out layer	Drop out value = 0.2	
Maximum pooling layer 1D	Pool size = 4	
Flatten layer	Layer size = default	
Dense layer	Layer size = 100	Activation = layer
Output classification dense layer	Layer size = 1	Activation function = sigmoid

- **Model Hyperparameters**

**Table 13:** Model Hyperparameters.

Hyperparameters	Properties
Epochs	25
Batch size	32
Learning rate	0.001
Loss	Binary cross entropy loss

**Figure 11:**
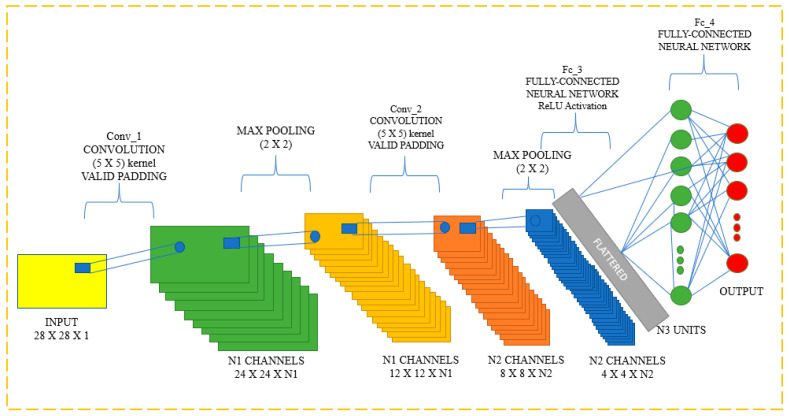
Classification Layer for Convolution Neural Network.

Input, output, and hidden layers are all features of CNNs that aid in the processing and classification of pictures. Convolutional, pooling, ReLU, and fully linked layers are included in the hidden layers. The CNN Classification layer is displayed in Figure 11.

Multiple artificial neuronal layers make up CNN. Artificial neurons are mathematical processes that compute the weighted sum of a number of inputs and output an activation value, just like their biological counterparts do. Each layer of a ConvNet generates several activation functions in response to the entry of a picture, which are subsequently transmitted to the following layer. Basic elements, including borders with a horizontal or diagonal axis, are often removed in the first layer. The layer below receives this output and thus can identify more complex properties, such as corners and multiple edges. The classification layer provides a series of confidence ratings (numbers between 0 and 1) that indicate how likely it is for the image to belong to a “class,” based on the activation map of the final convolution layer.

### 4.6. Model Training Results

The sklearn library is used to import both models. Both models classify each MDD with an accuracy of more than 80%. Table 14 and Table 15 show the outcomes of the two models.

- **Standard Deviation Score**

**Table 15:** Standard Deviation Score.

Std_Score
0.086534
0.078554
0.109268
0.121718
0.152891
0.136123
0.189414
0.088103
0.103762
0.074632
0.202616
0.189725

### 4.7. Model Evaluation

The MODMA dataset is taken into consideration in order to predict depression using EEG signals from MDD patients and healthy control individuals. First, linear characteristics and nonlinear features are retrieved from the EEG signals. Additionally, MODMA offers data from patients, including demographic and psychological assessment data. After that, the features are integrated with the demographic information, which includes details such as gender, age, and MDD type. MDD is divided into six classes.

- Obsessive-compulsive disorders;
- Addictive disorder;
- Trauma and stress-related disorder;
- Mood disorder;
- Schizophrenia;
- Anxiety disorder.

Every class has an additional two characteristics, namely patients with disorders and healthy controls. In model training, classes are used as the responses and the features that were extracted as predictors. In order to determine the accuracy of three distinct models for six different illnesses, models are then assessed using the testing dataset.

- **Evaluation of Training Model**

The following metrics are being considered for evaluation of the trained model

○ Accuracy score;
○ Micro F1 score;
○ Macro F1 score;
○ ROC curve;
○ Micro Recall score;
○ Macro recall score;
○ Macro precision score;
○ Micro precision score.

**Accuracy:** Ratio of the number of correct predictions to the total number of predictions, and this represents how often the classifier makes the correct predictions.

(1) $$Accuracy=\frac{TN+TP}{TN+FP+TP+FN}$$

Here, Equation (1) relates to an equation for accuracy, which expresses the proportion of correctly classified data instances to all other data instances.

If the dataset is unbalanced, accuracy might not be an acceptable metric (both negative and positive classes have different numbers of data instances).

**Precision:** Proportion of anticipated positives that are actually positive.

(2) $$Precision=\frac{TP}{TP+FP}$$

The precision model is shown in Equation (2). A good classifier’s precision should preferably be 1 (high). Only when the numerator and denominator are equal, or when TP = TP + FP, does precision become 1, which also implies that FP is zero. The accuracy value drops as FP rises because the denominator value exceeds the numerator.

**Recall:** The fraction of true positives successfully identified.

(3) $$Recall=\frac{TP}{TP+FN}$$

The recall equation is shown in Equation (3), where recall for a good classifier should ideally be 1 (high). Only when the numerator and denominator are identical, as in TP = TP + FN, does recall become 1, which also implies that FN is zero. As FN increases, the denominator value rises above the numerator and the recall value falls.

**F1 score:** The harmonic mean of recall and precision.

(4) $$F1\;Score=2*\frac{Precision*Recall}{Precision+Recall}$$

The F1 Score equation is shown in Equation (4). When precision and recall are both 1, the F1 Score is 1. Only when precision and recall are both strong can the F1 score rise. A more useful metric than accuracy is the F1 score, which is the harmonic mean of recall and precision. The results are as shown in the below Table 16.

## 5. Results

1D CNN Results

**Table 16:** One-dimensional CNN results.

loss:	0.3229 accuracy:	0.8790
loss:	0.2638 accuracy:	0.9004
loss:	0.2198 accuracy:	0.9217
loss:	0.2054 accuracy:	0.9004
loss:	0.1907 accuracy:	0.9253
loss:	0.1301 accuracy:	0.9573
loss:	0.1079 accuracy:	0.9644
loss:	0.1067 accuracy:	0.9644
loss:	0.0661 accuracy:	0.9858
loss:	0.0589 accuracy:	0.9858
loss:	0.0501 accuracy:	0.9929
loss:	0.0659 accuracy:	0.9822
loss:	0.0427 accuracy:	0.9929

**Figure 12:**
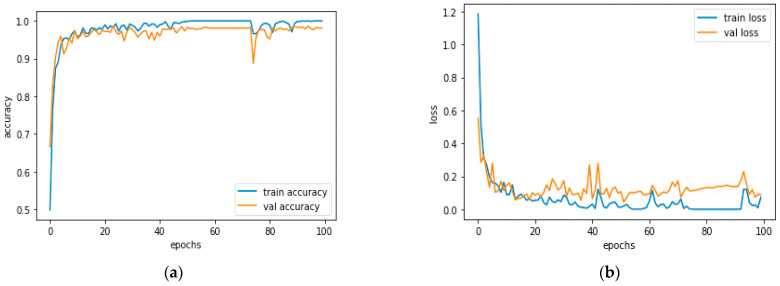
(**a**) Training Performance Graph; (**b**) Loss Performance Graph.

**Figure 13:**
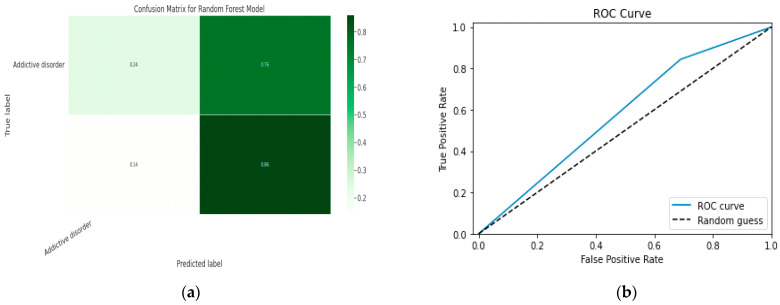
(**a**) Confusion Matrix for RF Model; (**b**) ROC Curve for RF Model.

Figure 12a and Figure 13a, which display the training performance graph and loss performance graph, are plotted in the sample below.

### 5.1. First Class Addictive Disorder

**Figure 14:**
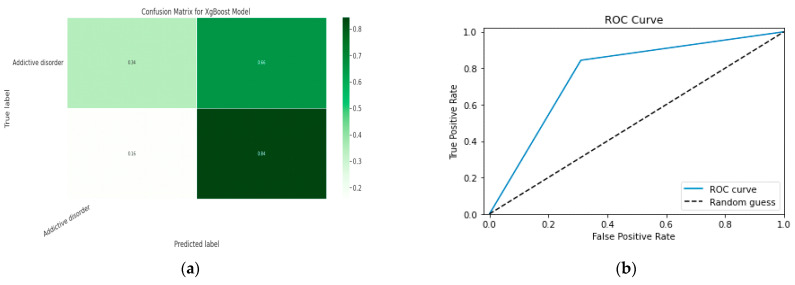
(**a**) Confusion Matrix for XGBoost Model; (**b**) ROC Curve for XGBoost Model.

Early detection of depression symptoms is a crucial initial step towards evaluation, diagnosis, and behavior modification. The performance of a classification model is determined using an N × N matrix termed a confusion matrix, where N is the total number of target classes. The RF model’s parameters are listed in Table 17 below. In comparison, the confusion matrix for the RF model is shown in Figure 14.

#### 5.1.1. Random Forest Classification Model

The model evaluation parameters for the RF classifier model are displayed in Table 17. Additionally, Figure 13a displays the RF classifier model’s confusion matrix.

#### 5.1.2. XGBoost classification Model

XGBoost with its traditional classifier will be the first algorithm we employ. This is the standard basic algorithm from the XGBoost library, and Table 18’s display of the XGBoost model’s parameters illustrates this. The confusion matrix for the XGBoost model is shown in Figure 14a.

Figure 14b shows the ROC graph for the XGBoost model. There are two linear graphs showing ROC curve and random curve

#### 5.1.3. CNN Classification Model

**Figure 15:**
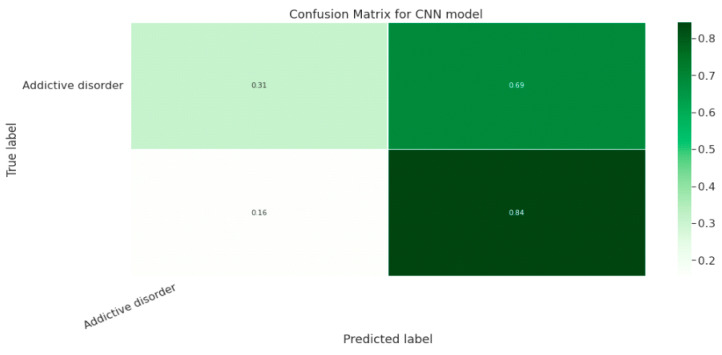
Confusion Matrix for CNN Classification Model.

The effectiveness of the categorization approach is summarized in a confusion matrix. In other words, the confusion matrix summarizes how well the classifier performed. The parameters of the CNN Classification model are shown in Table 19. The confusion matrix for the CNN model is shown in contrast in Figure 15.

**Figure 16:**
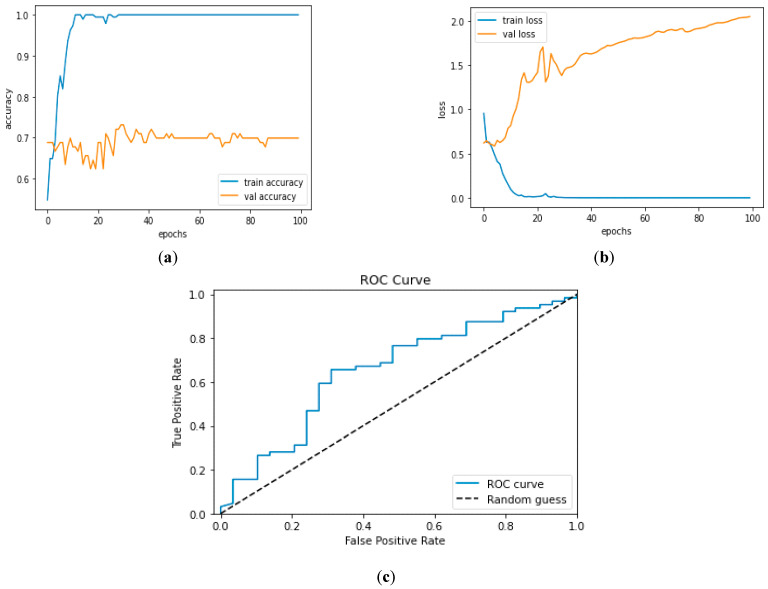
(**a**) Training and Validation Accuracy Performance Graph; (**b**) Training and Validation Loss Performance Graph; (**c**) ROC Curve for the CNN Classification Model.

Figure 16a shows the training and validation accuracy performance graph, where the blue graph shows the training accuracy and the orange graph shows the validation accuracy. Similarly, Figure 16b shows the training and validation loss performance graph. Figure 16c shows the ROC curve for the CNN Classification model.

#### 5.1.4. Class Obsessive-Compulsive Disorder

The broad category of neurotic, stress-related, and somatoform disorders, which also includes hypochondriacal disorder as a sub-group of somatoform disorders, is where OCD is categorized in the ICD-10.

### 5.2. Model Classification

#### 5.2.1. Random Forest Classifier Model

**Figure 17:**
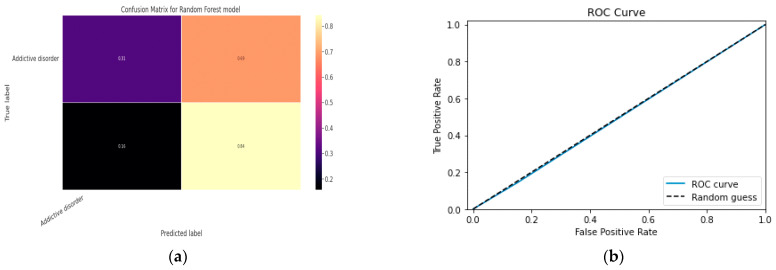
(**a**) Confusion Matrix of RF Classifier Model; (**b**) ROC curve for the RF Model.

Random Forest is an ensemble classifier made up of several Decision Trees that produces a class based on the average output of the class from each individual tree. The RF classifier model’s parameters are displayed in Table 20. Figure 17 depicts the RF classifier model’s confusion matrix in contrast.

Figure 17 shows the ROC curve for the RF classifier model. There are two linear graphs showing ROC curve and random curve.

#### 5.2.2. XGBoost Classifier Model

**Figure 18:**
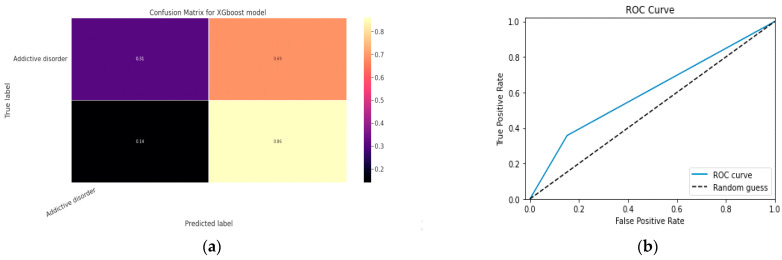
(**a**) Confusion Matrix of XGBoost Model; (**b**) ROC Curve for the XGBoost Model.

Table 21 displays the XGBoost model’s parameters. Figure 18a depicts the XGBoost model’s confusion matrix in contrast.

Figure 18 shows the ROC curve for the XGBoost classifier model. There are two linear graphs showing ROC curve and random curve.

#### 5.2.3. CNN Model

**Figure 19:**
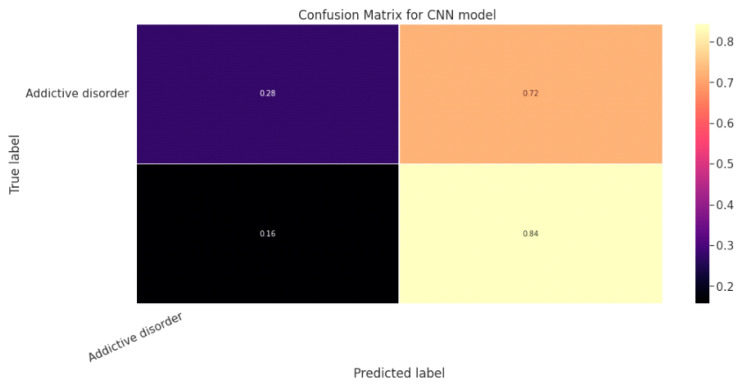
Confusion Matrix of CNN Model.

Table 22 lists the CNN model’s parameters, whereas Figure 19 shows the confusion matrix.

**Figure 20:**
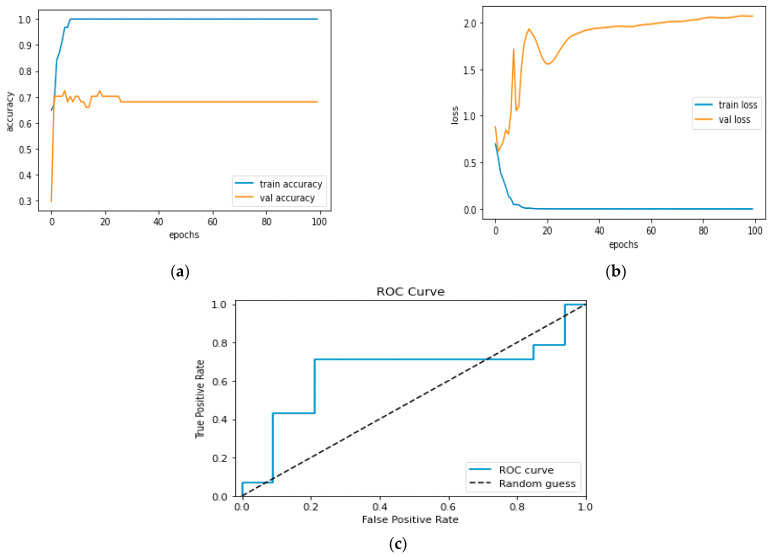
(**a**) Training and Validation Accuracy Performance Graph; (**b**) Training and Validation Loss Performance Graph; (**c**) ROC Curve.

Figure 20a shows the training and validation accuracy performance graph. The blue graph shows the training accuracy and the orange graph shows the validation accuracy. Similarly, Figure 20b shows the training and validation loss performance graph. Figure 20c shows the ROC curve for the CNN Classification model.

### 5.3. Class Trauma Stress-Related Disorder

#### 5.3.1. Random Forest Classifier

**Figure 21:**
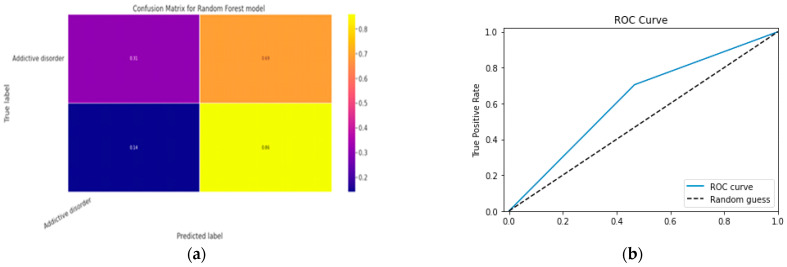
(**a**) Confusion Matrix of RF Classifier Model; (**b**) ROC Curve for the RF Model.

The model evaluation parameters for the RF classifier model are displayed in Table 23. Additionally, Figure 21a displays the RF classifier model’s confusion matrix.

Figure 21b shows the ROC curve for the RF classifier model. There are two linear graphs showing ROC curve and random curve.

#### 5.3.2. XGBoost Model

**Figure 22:**
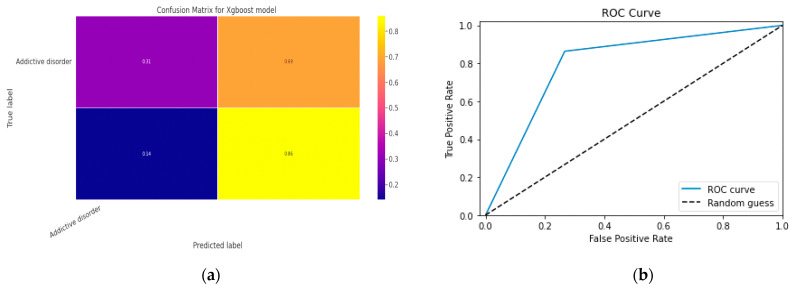
(**a**) Confusion Matrix of XGBoost Model; (**b**) ROC Curve for XGBoost Model.

Table 24 lists the XGBoost model’s parameters according to the Class Trauma Stress-related Disorder, and Figure 22 displays the confusion matrix.

Figure 22b shows the ROC curve for the XGBoost classifier model. There are two linear graphs showing ROC curve and random curve.

#### 5.3.3. CNN Model

**Figure 23:**
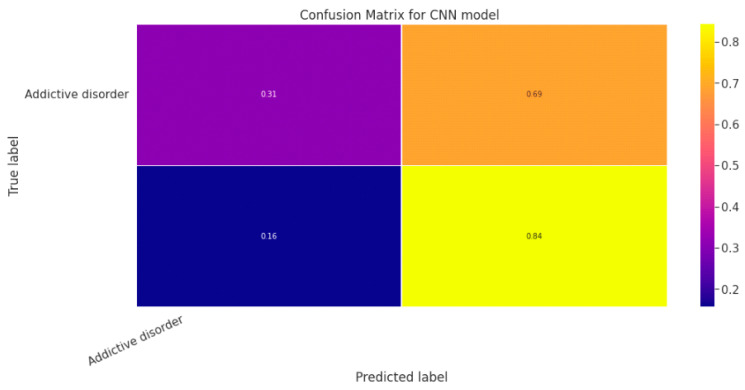
Confusion Matrix of CNN Model.

Table 25 lists the CNN model’s parameters, while Figure 23 depicts the confusion matrix for the same model.

**Figure 24:**
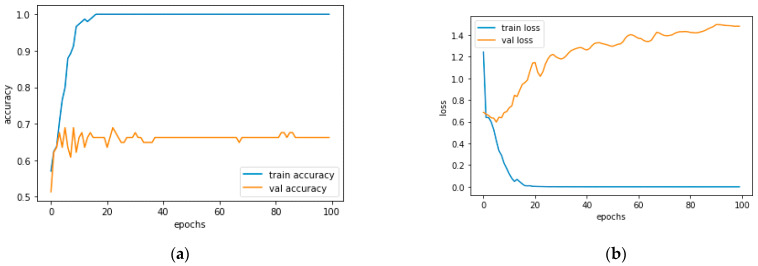

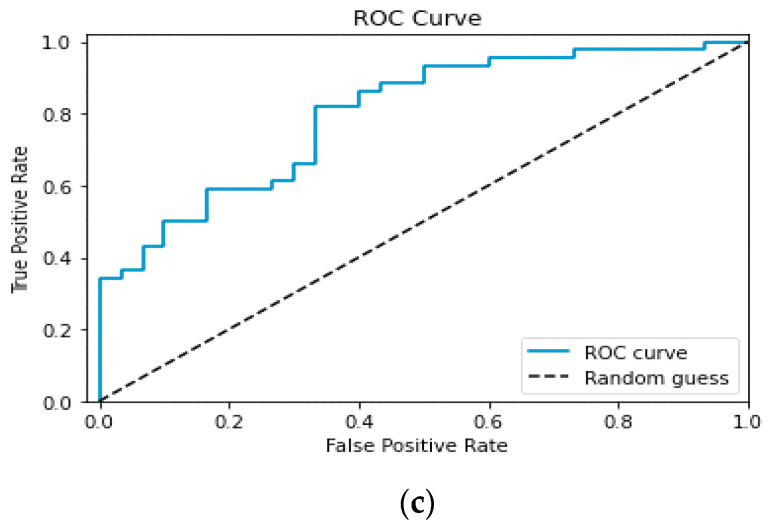
(**a**) Training and Validation Accuracy Performance Graph; (**b**) Training and Validation Loss Performance Graph; (**c**) ROC Curve.

Figure 24a shows the training and validation accuracy performance graph. The blue graph shows the training accuracy and the orange graph shows the validation accuracy. Similarly, Figure 24b shows the training and validation loss performance graph. Figure 24c shows the ROC curve for the CNN Classification model.

### 5.4. Class Mood Disorder

To identify mood disorders, we employ tree-based classification algorithms, specifically classification trees, along with the Random Forest, XGBoost, and CNN approaches.

#### 5.4.1. Random Forest Classifier

**Figure 25:**
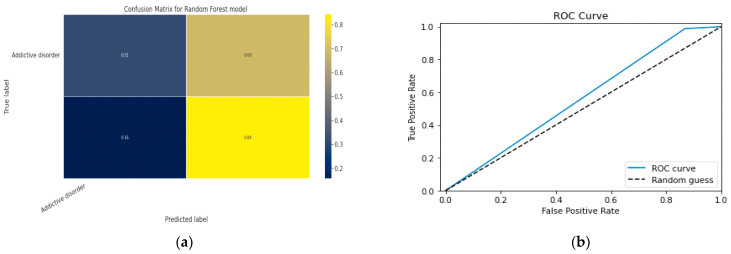
(**a**) Confusion Matrix of RF Model; (**b**) ROC Curve for the RF Model.

Figure 25a depicts the RF model’s confusion matrix, while Table 26 displays the model’s parameters.

Figure 25b shows the ROC curve for the RF classifier model. There are two linear graphs showing ROC curve and random curve.

#### 5.4.2. XGBoost Model

**Figure 26:**
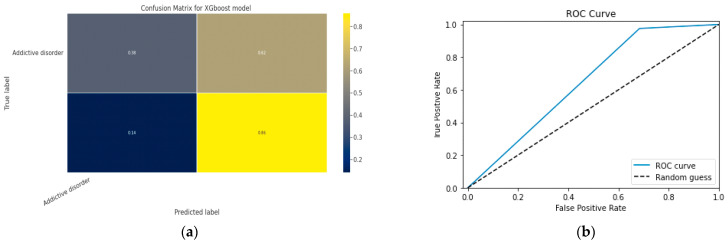
(**a**) Confusion Matrix of XGBoost Model; (**b**) ROC Curve for XGBoost Model.

Figure 26a depicts the XGBoost confusion matrix, while Table 27 displays the XGBoost model’s parameters.

Figure 26b shows the ROC curve for the XGBoost classifier model. There are two linear graphs showing ROC curve and random curve.

#### 5.4.3. CNN Model

**Figure 27:**
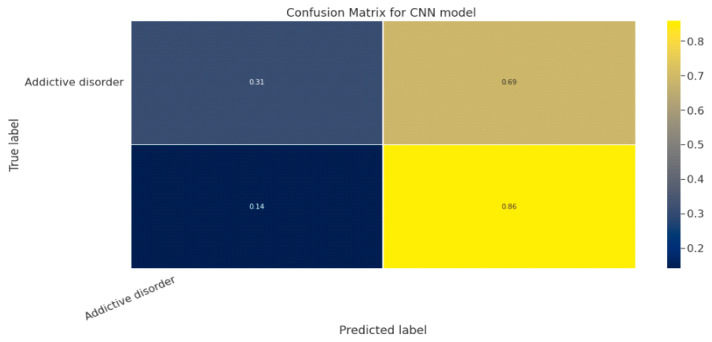
Confusion Matrix of CNN Model.

The parameters of the CNN model are shown in Table 28, while the CNN model’s confusion matrix is shown in Figure 27.

**Figure 28:**
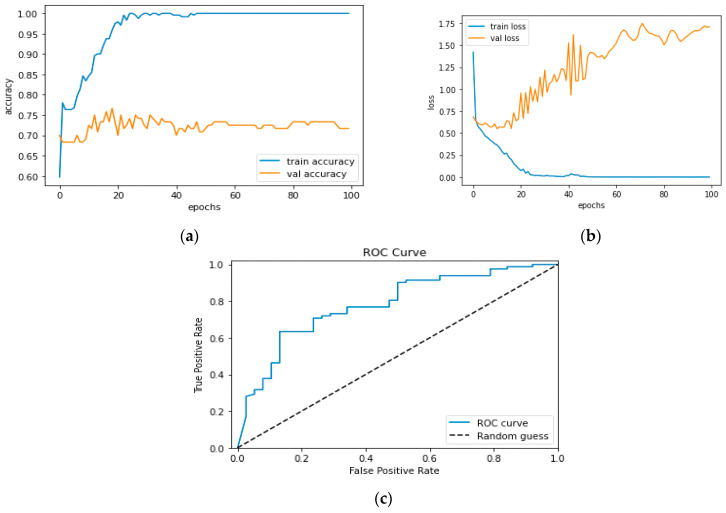
(**a**) Training and Validation Accuracy Performance Graph; (**b**) Training and Validation Loss Performance Graph; (**c**) ROC Curve.

Figure 28a shows the training and validation accuracy performance graph. The blue graph shows the training accuracy and the orange graph shows the validation accuracy. Similarly, Figure 28b shows the training and validation loss performance graph. Figure 27c shows the ROC curve for the CNN Classification model.

### 5.5. Class Schizophrenia

ML algorithms can identify diseases like schizophrenia and support clinical decision-making with predictive models. In order to forecast the presence of hospitalized schizophrenia patients, machine learning techniques such as Decision Tree, Random Forest, XGBoost, and CNN are used.

#### 5.5.1. Random Forest Model

**Figure 29:**
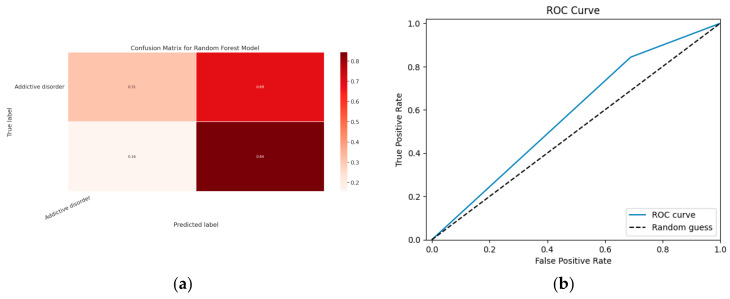
(**a**) Confusion Matrix of RF Model; (**b**) ROC Curve for RF Model.

Table 29 provides the RF model’s parameters according to Class Schizophrenia, and Figure 29a depicts the model’s confusion matrix.

Figure 29b shows the ROC curve for the RF classifier model. There are two linear graphs showing ROC curve and random curve.

#### 5.5.2. XGBoost Model

**Figure 30:**
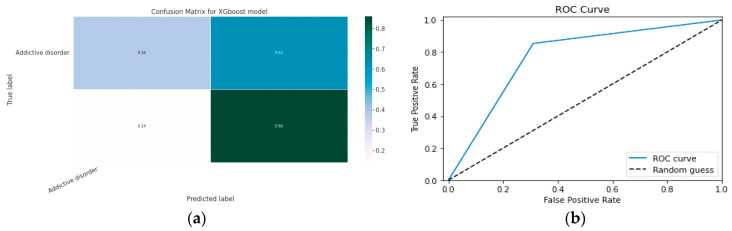
(**a**) Confusion Matrix of XGBoost Model; (**b**) ROC Curve for XGBoost Model.

The XGBoost parameters are shown in Table 30, and Figure 30a provides the confusion matrix.

Figure 30b shows the ROC curve for the XGBoost classifier model. There are two linear graphs showing ROC curve and random curve.

#### 5.5.3. CNN Model

**Figure 31:**
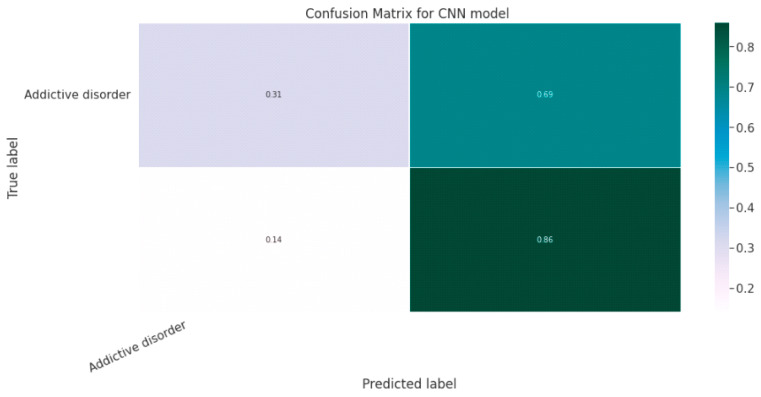
Confusion Matrix of CNN Model.

Table 31 lists the CNN model’s parameters, and Figure 31 displays the model’s confusion matrix.

**Figure 32:**
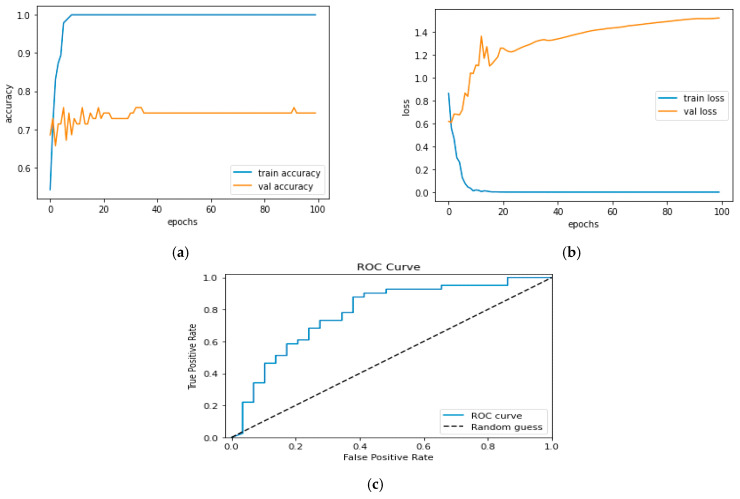
(**a**) Training and Validation Accuracy Performance Graph; (**b**) Training and Validation Loss Performance Graph; (**c**) ROC Curve.

Figure 32a shows the training and validation accuracy performance graph. The blue graph shows the training accuracy and the orange graph shows the validation accuracy. Similarly, Figure 32b shows the training and validation loss performance graph. Figure 31c shows the ROC curve for the CNN Classification model.

### 5.6. Anxiety Disorder

Data mining is able to find hidden patterns and associations that can be utilized to forecast generalized anxiety disorder, which leads to substantial insights. The Random Forest approach is one of the categorization data mining strategies that embeds good predictive properties for accurate prediction.

#### 5.6.1. Random Forest Classifier Model

**Figure 33:**
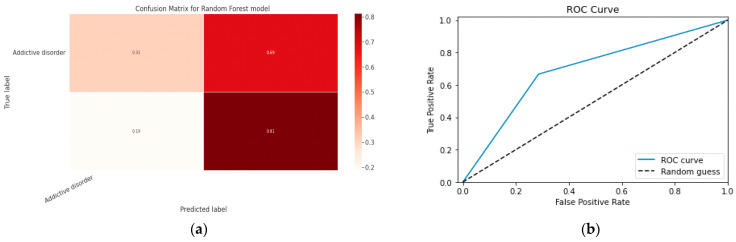
(**a**) Confusion Matrix of RF Model; (**b**) ROC Curve for RF Model.

Figure 33a depicts the RF model’s confusion matrix, while Table 32 displays the parameters for the RF classifier model.

Figure 33b shows the ROC curve for the RF classifier model. There are two linear graphs showing ROC curve and random curve.

#### 5.6.2. XGBoost Model

**Figure 34:**
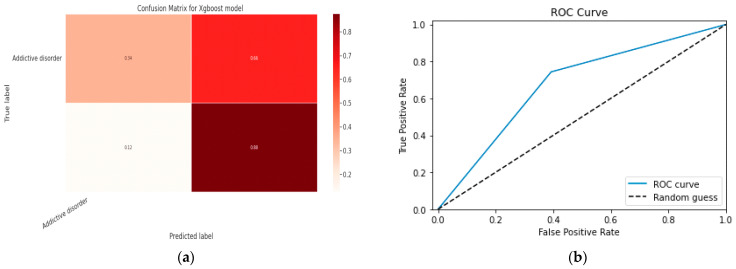
(**a**) Confusion Matrix of XGBoost Model; (**b**) ROC Curve for XGBoost Model.

The XGBoost model’s parameters are listed in Table 33, and Figure 34a provides the model’s confusion matrix.

Figure 34b shows the ROC curve for the XGBoost classifier model. There are two linear graphs showing ROC curve and random curve.

#### 5.6.3. CNN Model

**Figure 35:**
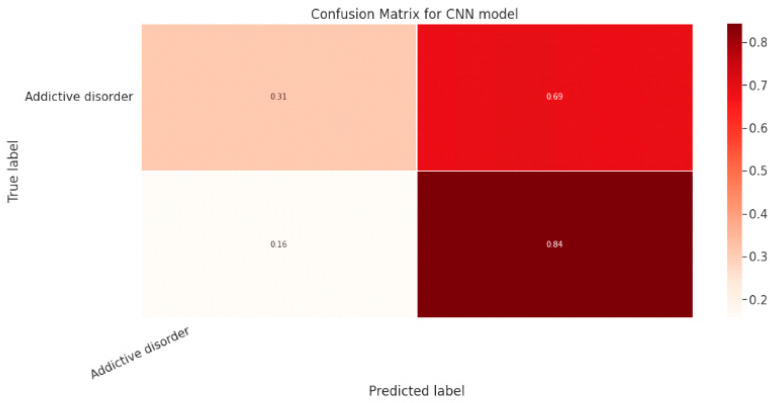
Confusion Matrix of CNN Model.

Table 34 displays the CNN model’s parameters, and Figure 35 displays the CNN model’s confusion matrix.

**Figure 36:**
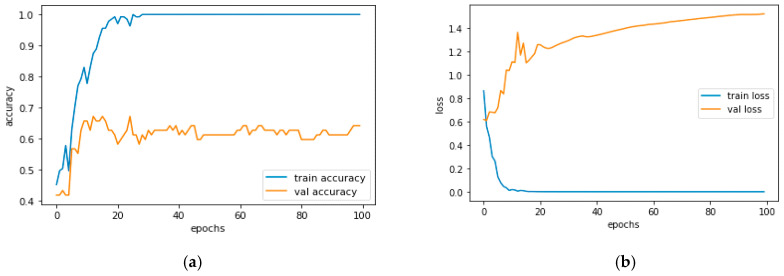

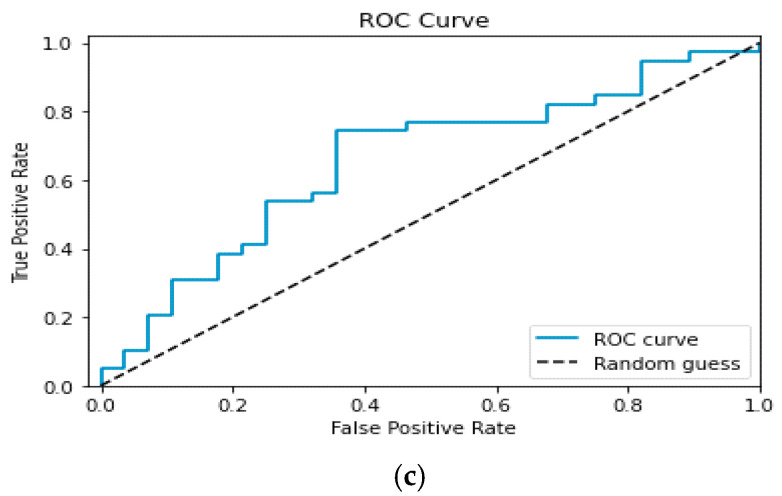
(**a**) Training and Validation Accuracy Performance Graph; (**b**) Training and Validation Loss Performance Graph; (**c**) ROC Curve.

Figure 36a shows the training and validation accuracy performance graph. The blue graph shows the training accuracy and the orange graph shows the validation accuracy. Similarly, Figure 36b shows the training and validation loss performance graph. Figure 36c shows the ROC curve for the CNN Classification model.

## 6. Discussion

Electroencephalograms serve as an important point of reference and an objective foundation for the detection and diagnosis of depression (EEGs). In order to improve the diagnostic accuracy, a high-performance hybrid neural network depression detection strategy using deep learning technology is proposed in this research. This research considers resting-state neurological signals via 128 channels. Data from both an advanced wearable EEG collector and a traditional 128-electrode elastic cap are given. Results for categorization accuracy from three models were inconsistent. When comparing CNN to the other two models, it performs data classification more accurately. EEG signal data are collected from the multi-modal open dataset MODMA, which is employed in the study of mental diseases. The EEG dataset contains information from both a traditional 128-electrode elastic cap and a cutting-edge wearable 3-electrode EEG collector for widespread applications. There are three datastores for EEG. An EEG machine typically has a lot of electrodes. After obtaining voltage from the patient’s scalp, the electrodes convert the voltage into signal data. For instance, each electrode will generate a time series of voltage values if there are n electrodes. The voltage in different areas of the brain varies.

A bandpass filter with a 50 Hz filter size is used to remove the power spectral distribution spikes. The programme not only removes these spikes but also converts the EEG signal into a NumPy array since we cannot analyze signals directly for feature extraction. The EEG signals are processed to reduce noise and converted to a NumPy array, and feature engineering is applied to the data to extract useful features that will be used to differentiate between the EEG power spectrum of a healthy individual and that of a mentally ill person. We extract spectral entropy and singular-value deposition entropy, two nonlinear characteristics, from the provided EEG signal datastore. The signal loses a lot of important information, as evidenced by these two properties. A number of patients’ resting EEG data were first used to derive characteristics. These statistics are now combined with details about the patient’s condition and demographics. The gathered qualities will be used as a predictor, and the patient’s current state of health will be the response. The two fundamental groups into which the patient’s state can be separated are major depressive disorder and healthy control. There are six additional MDD subtypes: Mental illnesses such as, for example, obsessive-compulsive disorders, addiction disorders, disorders linked to trauma and stress, mood disorders, schizophrenia, and anxiety disorders.

In this study, three machine learning models—Random Forest, XGBoost, and CNN-based models—are used to analyze MODMA data in order to diagnose depression. The study’s objective is to identify traits and link those qualities to the appropriate labels—in this case, MDD and healthy controls. We translate these labels to 1 and 0. There are six major depressive illnesses that fall under the MDD umbrella. Models will be trained using attributes and particular labels from the MDD class. Three models produced results for categorization accuracy that varied. Comparing CNN to the other two models, it is more accurate in classifying data. CNN reported a 97% accuracy rate for training with 25-epoch iterations.

The suggested strategy offers a number of advantages. First, it can more accurately identify between persons with depression and healthy participants based on the same dataset than previous methods. The network model also includes an attention mechanism that considerably reduces training time. The results show that by focusing computing resources on traits with high weights, the attention mechanism decreases overhead.

### Comparative Analysis

Three key classification techniques—Xgboost, Random Forest, and CNN model—are used in the design of our model. The best accuracy, 97%, was provided by the CNN model over 25 training epochs. With a large number of layers, the CNN model is designed to learn data trends with greater precision. In EEG diagnosis of depression based on multi-channel data fusion and clipping augmentation and convolutional neural network, a maximum accuracy of 90.02 is attained with the aid of the CNN model as compared to some prior work for depression identification with the use of the MODMA dataset. Using the CNN + GRU model, the minimum accuracy in comparable work is achieved of up to 89.63. In our model, we employed a lower learning rate and a greater number of epochs, which assisted the CNN model in extracting the greatest number of features from the dataset and providing the most accuracy. Table 35 lists some of the most recent works. Using a Deep Learning CNN network, our technique had a 97% accuracy rate.

There are a number of useful aspects of the suggested model for EEG-based depression detection using multiple ML techniques that could make it useful in practical applications. The proposed model’s capacity to increase diagnostic precision for depression by fusing EEG signals with demographic information such as age and gender is one of its main benefits. Earlier and more efficient treatment might result from this, which would ultimately improve patient outcomes. The proposed model’s use of open datasets, particularly the MODMA dataset for gathering EEG signal data, is another practical feature. This broadens the information available for studying mental illnesses and improves the approach’s usability and applicability in the real world. This method is effective and reliable, automating the diagnosis of depression through the use of machine learning and deep learning techniques for automatic depression detection from EEG signals. This may lessen the amount of work clinicians have to do and increase the speed and precision of diagnosis. Furthermore, the proposed model can be applied widely with both conventional 128-electrode elastic caps and cutting-edge wearable 3-electrode EEG collectors. This makes data collection more flexible and convenient, increasing its accessibility to a wider range of patients.

## 7. Conclusions

In order to comprehensively examine the features of EEG signals and recommend a high-performance technique for mental state detection, the researchers used DL algorithms as the study object and EEG signals as the research object. After that, the model’s model parameters and hyperparameters were adjusted via testing; comparison studies were conducted to confirm the approach’s applicability and effectiveness. The approach indicated in this study is more productive in terms of recognizing and diagnosing depression, based on the trial data. In the case of a few repetitions, our algorithm might dynamically extract the EEG signal features to outperform earlier methods in classification performance. This method is implemented well on open datasets and establishes a technological foundation for the evaluation and diagnosis of depression. The performance and effectiveness of the methodology were validated through comparative experiments. The approach utilized in this research has a 98% accuracy rate when applied to the public dataset.

Even if the model is successful in identifying mental states, the following problems need to be fixed: Despite the fact that there were not enough negative samples, the dataset used in this research can substantiate the observations made in this article. In the future, we intend to gather more diagnostic data to enhance the generalization ability of the model. Additionally, as the main objective of this research was to diagnose depression, future studies on non-destructive treatments could be taken into consideration. Heavy electrode caps that had to be forced on the scalp surface in order to fully connect with them were employed to capture EEG data from the study’s participants. As a result, some users might have felt pain. We can take into consideration employing fewer, lighter electrodes in future studies with portable acquisition techniques such as ear-BCI. (3) Only a qualitative analysis of the psychological state was carried out in this investigation. Future study might involve quantitative assessments of psychological status. Depression may be diagnosed depending on its intensity, which is further classified as normal, mild, moderate, or severe. The functional form could be established as a confirmation to exhibit the concentration level. When diagnosing depression, it is beneficial to consider demographic factors other than age and gender, like ethnicity and socioeconomic status. These elements may significantly affect the prevalence and severity of depression, but our paper did not specifically address them. Future studies can be carried out that take these factors into account.

## Figures and Tables

**Table 1:** List of Earlier Research Publications Cited Along with Their Methodologies and Findings.

Ref.	Methodology	Experimental Parameters/Dataset	Libraries/Platform Implementation	Outcome/Results	Main Focus
[1]	Depression, depressive disorders, EEG, CNN, and LSTM	Deep learning techniques have been employed to identify or predict depression. What methods are primarily employed for feature extraction from EEG signals?	FFT, CNN, 1DCNN, 2DCNN, and 3DCNN	Using convolutional layers end-to-end, local features were extracted.	DL is used to diagnose depression using EEG readings.
[2]	CNN, RNN, RNN with LSTM, DL, ML, MLP	When 40% of the testing set’s data are present, RNN with LSTM model is used.	SVM and Neural Network-Based DL	Two supervised ML models, SVM and LR, outperformed each other with accuracy rates of about 97.85 percent in testing and 100% in training, respectively.	A Comparative Study Using DL to Monitor Mental Depression Using EEG Data
[4]	EEG; Clinical depression	Convolution layers are convolved with the input signal to produce feature maps.	LSTM Model, CNN	Using the random splitting method, the model was tested, and the results showed 99.23% and 99.05% accuracy for the right and left hemispheres’ EEG signals, respectively.	Automatic clinical depression detection
[6]	EEG, CNN, Transfer Learning	Visual abstract theta, alpha, and beta band EEG power is calculated.	CAD; ConvNet	The proposed system delivered an 85.82% accuracy rate.	CNN’s use for recognizing mild depression
[7]	EEG data, SVM, LR, and LNR are associated with MDD.	Ratio of features taken out of EEG signals in different frequency bands.	Elimination of recursive features, Pearson correlation coefficient	The development of this MDD detection framework may be integrated into a healthcare system to assist medical professionals in identifying MDD patients.	Framework for detecting depressive disorders with two stages of feature selection
[8]	Symptoms of child anxiety related to the Children’s Depression Inventory	Sample of 451 young adults and adolescents.	Multivariable linear regression	There was an increase in depression and somatic/panic symptoms in females, in addition to social anxiety and social phobia.	Symptoms of anxiety
[37]	Decision Tree, Variance, SVM, and Feature Selection	13 features in total were retrieved, and a subset of the 6500 total features was calculated.	RF Model, FDR-based feature selection, and tree-based feature selection	Calculations of the linear, non-linear, and power spectral features were made for each channel of the EEG data for each sub-band.	Using ML, an EEG-Based Depression Detection Method
[11]	ANN, DL, DNN, FFNN Network, EEG, MDD	Trans diagnostic cohorts.	EEG Data + Computational Tool + MATLAB	It has a classification accuracy rate of 97.66%.	Utilizing neural networks to detect bipolar disorder

**Table 2:** MODMA Dataset description.

Experiment Type	Recording Type	Number of Participants	Outpatients (M/F)	Healthy Controls (M/F)	Age Range
When External Stimulation is Used	128-channel event-related potential recordings	53	13/11	20/9	16–56 years
Three channels when at rest	3-channel resting-state recordings	55	15/11	19/10	16–56 years
Under Rest	128-channel recordings	53	16/8	20/9	16–56 years

**Table 3:** Linear Features.

	if_alpha_one_pat	if_beta_one_pat	if_delta_one_pat	if_theta_one_pat	if_mean_one_pat	if_max_one_pat	if_min_one_pat	if_median_one_pat
E9	1875.276458	549.464641	2901.380098	1367.535960	18,576.888899	2.132937 × 10^7^	21.852823	600.045377
E22	1962.344324	564.344696	2103.001899	1128.255877	5045.360363	4.711530 × 10^6^	11.292816	582.253467
E24	1829.038390	500.105839	1683.582830	920.839226	19,974.846182	2.365145 × 10^7^	27.258894	528.262270
E33	1762.225038	655.299494	3190.643405	1483.650170	21,054.315858	2.431015 × 10^7^	33.866445	662.574884
E36	1290.414086	442.475702	1464.350040	821.077427	10,529.862183	1.211437 × 10^7^	11.256188	428.169135

**Table 4:** Non-Linear Feature Extraction.

	nl_svden_one_pat	nl_spec_enone_pat	nl_permenone_pat
E9	0.460683	0.385432	0.781321
E22	0.460976	0.423530	0.769295
E24	0.456698	0.379321	0.778964
E33	0.452907	0.395446	0.779078
E36	0.460703	0.450232	0.770696

**Table 5:** Data Frame for Feature Datastore.

	if_alpha_resting_E36	if_beta_resting_E36	if_delta_resting_E36	if_theta_resting_E36	if_mean_resting_E36	if_max_resting_E36	if_min_resting_E36
0	4884.613979	2278.624552	17,200.055374	6866.060694	89,740.273531	9.384269 × 10^8^	63.744193
1	4449.706266	2262.541526	19,716.190512	7544.042248	76,589.372973	7.777201 × 10^8^	96.528437
2	2535.166056	2705.244726	5962.754681	2638.860812	21,714.444838	2.003181 × 10^8^	5.312277
3	4719.376369	2299.826126	16,074.572713	6303.707905	48,324.405457	4.815979 × 10^8^	21.432547
4	7827.445436	3818.779974	33,842.519264	12,699.762977	71,679.363955	6.715888 × 10^8^	788.428172

**Table 6:** Demographic Information stored in the Pandas Data Frame.

	No.	Sex	Age	Eeg.Date	Education	IQ	Main.Disorder
0	1	M	57.0	2012.8.30	NaN	NaN	Addictive disorder
1	2	M	37.0	2012.9.6	6.0	120.0	Addictive disorder
2	3	M	32.0	2012.9.10	16.0	113.0	Addictive disorder
3	4	M	35.0	2012.10.8	18.0	126.0	Addictive disorder
4	5	M	36.0	2012.10.18	16.0	112.0	Addictive disorder
……	1	***	***	…	…	…	
940	941	M	22.0	2014.8.28	13.0	116.0	Healthy control
941	942	M	26.0	2014.9.19	13.0	118.0	Healthy control
942	943	M	26.0	2014.9.27	16.0	113.0	Healthy control
943	944	M	24.0	2014.9.20	13.0	107.0	Healthy control
944	945	M	21.0	2015.10.23	13.0	105.0	Healthy control

*** represents here a sequence of records. They can include all the records of the table.

**Table 7:** Data Frame.

	No.	Sex	Age	Eeg Date	Education	IQ	Main Disorder
0	1	M	57.0	2012.8.30	NaN	NaN	Addictive disorder
1	2	M	37.0	2012.9.6	6.0	120.0	Addictive disorder
2	3	M	32.0	2012.9.10	16.0	113.0	Addictive disorder
3	4	M	35.0	2012.10.8	18.0	126.0	Addictive disorder
4	5	M	36.0	2012.10.18	16.0	112.0	Addictive disorder
…				…	…		
940	941	M	22.0	2014.8.28	13.0	116.0	Healthy control
941	942	M	26.0	2014.9.19	13.0	118.0	Healthy control
942	943	M	26.0	2014.9.27	16.0	113.0	Healthy control
943	944	M	24.0	2014.9.20	13.0	107.0	Healthy control
944	945	M	21.0	2015.10.23	13.0	105.0	Healthy control

**Table 8:** Label Datastore.

{‘Additive disorder’: 0	1.00
1	1.00
2	1.00
3	1.00
4	1.00
……	
940	0.00
941	0.00
942	0.00
943	0.00
944	0.00
Name: main.disorder, length: 281, dtype: float64,	
trauma; and ‘stress related disorder’: 31	1.00
32	1.00
33	1.00
34	1.00
35	1.00
	………
940	0.00
941	0.00
942	0.00
943	0.00
944	0.00
Name: main.disorder, length: 223, dtype: float64,	
‘mood disorder’:89	1.00

**Table 9:** Feature Datastore.

{‘Addictive disorder’:			Sex	Age	Education	IQ	delta.FP1	delta.F7
0	0.00	4.04	13.00	102.00	3.58	3.08		3.07
1	6.60	3.62	6.00	120.00	2.60	2.40		2.4B6165
2	6.60	3.47	16.00	113.00	3.40	3.32		2.84
3	6.60	3.56	18.00	126.00	3.07	3.08		2.85
4	6.60	3.SB3S19	16.00	112.00	3.63	3.51		3.08
940	0.00	3.09	13.00	116.00	3.73	3.66		3.78
941	0.00	3.258B97	13.00	118.00	2. 943747	2.965345		3.317324
942	0.00	3.258B97	16.00	113.00	3.36	3.48		2.461167
943	0.00	3.18	13.00	107.00	2.992181	3.23		2.676375
944	0.00	3.04	13.00	105.00	4.17738B	4.24		3.565626
6	delta. F3 3.289336	delta. Fz 3.281344	delta. F4 3.247761		COH.gamma.Pz.P4 4.025159		COH.gamma.Pz.T6\ 2.817782	
1	2.73	2.649B26	2.52		3.82		2.86	
2	3.16	3.30	2.67		4.60		4.26	
3	2.63	2.65	2.57		4.09		4.16	
4	3.08	3.13	3.07		4.12		4.08	
940	3.61	3.21	3.16		4.417763		3.55	
941	3.01	2.97	3.01		4.188416		4.20	
					4.11		82.00	
943	2.80	2.87	2:733		4.59		3.88	

**Table 10:** Parameters for XGBoost Model.

Parameters	Values
Number of estimators	[100, 300, 500]
Sub-sample	[0.3, 0.5, 1]
Maximum depth of the tree	[1, 3, 6, none]

**Table 11:** Parameters of Random Forest Model.

Parameters	Values
Number of estimators	[100, 300, 500]
Maximum depth of the tree	[1, 3, 6, none]

**Table 14:** Model Training Results.

Disorder	Algorithm	Params	Mean_Score
Addictive disorder	RF	{‘max_depth’: None, ‘n _estimators’: 500}	0.788509
Addictive disorder	XGB	{‘max_depth“: 1, ‘n_estimators’: 100, ‘subsamp…	0.851462
Trauma and stress related disorder	RF	{‘max_depth’: None, ‘n_estimators’: 500}	0.826282
Trauma and stress related disorder	XGB	{‘max_depth“: 1, ‘n_estimators’: 100, ‘subsamp …	0.891538
Mood disorder	RF	{‘max_depth’: None, ‘n_estimators’: 500}	0.792669
Mood disorder	XGB	[‘max_depth“: 1, ‘n_estimators’: 500, ‘subsamp …	0.818229
Obsessive-compulsive disorder	RF	{‘max_depth’: 6, ‘n_estimators’: 100}	0.633889
Obsessive-compulsive disorder	XGB	{‘max_depth“: 3, ‘n_estimators’: 100, ‘subsamp …	0.689944
Schizophrenia	RF	{‘max_depth’: 3, ‘n_estimators’: 500}	0.808232
Schizophrenia	XGB	[‘max_depth’: 1, ‘n_estimators’: 100, ‘subsamp …	0.922694
Anxiety disorder	RF	{“max_depth’: None, ‘n_estimators’: 500}	0.759566
Anxiety disorder	XGB	(‘max_depth’: 1, ‘n_estimators’: 100, ‘subsamp …	0.828283

**Table 17:** Parameters of the RF Model.

Model	Accuracy	Micro F1 Score	Macro F1 Score	Micro Recall	Macro Recall	Micro Precision	Macro Precision
RF	0.82	0.760	0.84	0.88	0.89	0.91	0.91

**Table 18:** Parameters of the XGBoost Model.

Model	Accuracy Score	Micro F1 Score	Macro F1 Score	Micro Recall Score	Macro Recall Score	Micro Precision Score	Macro Precision Score
XGboost	0.85	0.81	0.86	0.91	0.93	0.92	0.93

**Table 19:** Parameters of the CNN Classification Model.

Model	Accuracy Score	Micro F1 Score	Macro F1 Score	Micro Recall Score	Macro Recall Score	Micro Precision Score	Macro Precision Score
CNN	0.94	0.95	0.90	0.92	0.94	0.91	0.95

**Table 20:** Parameters of RF Classifier Model.

Model	Accuracy Score	Micro F1 Score	Macro F1 Score	Micro Recall Score	Macro Recall Score	Micro Precision Score	Macro Precision Score
RF	0.79	0.82	0.81	0.86	0.81	0.83	0.88

**Table 21:** Parameters of XGBoost Model.

Model	Accuracy Score	Micro F1 Score	Macro F1 Score	Micro Recall Score	Macro Recall Score	Micro Precision Score	Macro Precision Score
XGboost	0.84	0.86	0.87	0.89	0.85	0.86	0.82

**Table 22:** Parameters of CNN Model.

Model	Accuracy Score	Micro F1 Score	Macro F1 Score	Micro Recall Score	Macro Recall Score	Micro Precision Score	Macro Precision Score
CNN	0.96	0.93	0.94	0.91	0.93	0.94	0.96

**Table 23:** Model Evaluation Parameters of RF Classifier Model.

Model	Accuracy Score	Micro F1 Score	Macro F1 Score	Micro Recall Score	Macro Recall Score	Micro Precision Score	Macro Precision Score
RF	0.73	0.78	0.81	0.82	0.84	0.83	0.89

**Table 24:** Parameters of XGBoost Model.

Model	Accuracy Score	Micro F1 Score	Macro F1 Score	Micro Recall Score	Macro Recall Score	Micro Precision Score	Macro Precision Score
XGBoost	0.82	0.79	0.84	0.85	0.81	0.83	0.88

**Table 25:** Parameters of CNN Model.

Model	Accuracy Score	Micro F1 Score	Macro F1 Score	Micro Recall Score	Macro Recall Score	Micro Precision Score	Macro Precision Score
CNN	0.91	0.90	0.96	0.94	0.93	0.98	0.95

**Table 26:** Parameters of RF Model.

Model	Accuracy Score	Micro F1 Score	Macro F1 Score	Micro Recall Score	Macro Recall Score	Micro Precision Score	Macro Precision Score
RF	0.76	0.78	0.71	0.75	0.81	0.83	0.84

**Table 27:** Parameters of XGBoost Model.

Model	Accuracy Score	Micro F1 Score	Macro F1 Score	Micro Recall Score	Macro Recall Score	Micro Precision Score	Macro Precision Score
XGboost	0.81	0.88	0.93	0.84	0.81	0.86	0.87

**Table 28:** Parameters of CNN Model.

Model	Accuracy Score	Micro F1 Score	Macro F1 Score	Micro Recall Score	Macro Recall Score	Micro Precision Score	Macro Precision Score
CNN	0.93	0.94	0.95	0.91	0.92	0.90	0.95

**Table 29:** Parameters of RF Model.

Model	Accuracy Score	Micro F1 Score	Macro F1 Score	Micro Recall Score	Macro Recall Score	Micro Precision Score	Macro Precision Score
RF	0.72	0.76	0.79	0.78	0.73	0.81	0.79

**Table 30:** Parameters of XGBoost Model.

Model	Accuracy Score	Micro F1 Score	Macro F1 Score	Micro Recall Score	Macro Recall Score	Micro Precision Score	Macro Precision Score
boost	0.80	0.83	0.81	0.88	0.87	0.83	0.79

**Table 31:** Parameters of CNN Model.

Model	Accuracy Score	Micro F1 Score	Macro F1 Score	Micro Recall Score	Macro Recall Score	Micro Precision Score	Macro Precision Score
CNN	0.93	0.95	0.97	0.91	0.93	0.95	0.98

**Table 32:** Parameters of RF Classifier Model.

Model	Accuracy Score	Micro F1 Score	Macro F1 Score	Micro Recall Score	Macro Recall Score	Micro Precision Score	Macro Precision Score
RF	0.73	0.72	0.69	0.57	0.66	0.68	0.7

**Table 33:** Parameters of XGBoost Model.

Model	Accuracy Score	Micro F1 Score	Macro F1 Score	Micro Recall Score	Macro Recall Score	Micro Precision Score	Macro Precision Score
xgboost	0.77	0.75	0.78	0.81	0.83	0.84	0.86

**Table 34:** Parameters of CNN Model.

Model	Accuracy Score	Micro F1 Score	Macro F1 Score	Micro Recall Score	Macro Recall Score	Micro Precision Score	Macro Precision Score
CNN	0.94	0.97	0.98	0.91	0.95	0.98	0.99

**Table 35:** List of a few Recent Works.

Previous Work	Approach	Accuracy
[38]	CNN + GRU	89.63
[39]	CNN	91.01
[40]	ResNet-50 + LSTM	90.02
Proposed	CNN network	97%

## Data Availability

Dataset is available on request.
